# Study protocol: The Dutch 20|30 Postmeningitis study: a cross-sectional follow-up of two historical childhood bacterial meningitis cohorts on long-term outcomes

**DOI:** 10.1186/s12887-019-1900-1

**Published:** 2019-12-30

**Authors:** O. El Tahir, R. C. J. de Jonge, S. Ouburg, S. A. Morré, A. M. van Furth

**Affiliations:** 1 Department of Pediatric Infectious Diseases and Immunology, AI&II, Amsterdam UMC, Vrije Universiteit Amsterdam, Amsterdam, The Netherlands; 2 Department of Pediatric Surgery, Erasmus MC Rotterdam - Sophia Children’s Hospital Pediatric Intensive Care Unit, Rotterdam, The Netherlands; 3 Department of Medical Microbiology and Infection Control, Laboratory of Immunogenetics VU University Medical Center, Amsterdam, The Netherlands; 4 Department of Genetics and Cell Biology, Institute for Public Health Genomics (IPHG), Research School GROW (School for Oncology & Developmental Biology), Faculty of Health, Medicine & Life Sciences, University of Maastricht, Maastricht, The Netherlands

**Keywords:** Bacterial meningitis, Children, Single nucleotide polymorphisms, Sequelae, Central nervous system, Infection, Hearing loss

## Abstract

**Background:**

Bacterial meningitis (BM) is a serious, life-threatening infectious disease of the central nervous system that often occurs in young children. The most common severe to moderate sequelae following BM are sensorineural hearing loss, neuromotor disabilities and mental retardation, while subtle sequelae include academic and behavioral disabilities. It is largely unknown whether these more subtle sequelae persist into adolescence and adulthood. Therefore, this study will investigate the very long-term effects of childhood BM in later life. Better understanding of long-term effects and early identification of adverse outcomes after BM are essential for more timely interventions. Additionally, certain single nucleotide polymorphisms (SNPs) are associated with disease severity and might predict adverse sequelae. These include SNPs in genes encoding for pathogen recognition and immune response upon infection. Accordingly, a secondary objective of this study is to investigate the role of genetic variation in BM and use any insights to predict short- and long-term outcomes.

**Methods:**

In the Dutch 20|30 Postmeningitis study, adolescents and young adults (*n* = 947) from two historical cohorts with a prior episode of BM during childhood will be enrolled into a cross-sectional follow-up investigation using mainly questionnaires that examine executive and behavioral functioning, health-related quality of life, subjective hearing, mood and sleeping disorders, academic performance, and economic self-sufficiency. The results will be compared to normative data by one-sample t-tests. Multivariable regression analysis will be used to assess for any associations with causative pathogens and severity of BM. Participants that complete the questionnaires will be approached to provide a swab for buccal DNA and subsequent sequencing analyses. Logistic regression models will be used to predict sequelae.

**Discussion:**

The unique follow-up duration of this cohort will enable us to gain insights into the possible very long-term adverse effects of childhood BM and how these might impact on quality of life. The investigation of host genetic factors will contribute to the development of prediction models which will serve as prognostic tools to identify children who are at high risk of adverse outcome after BM.

**Trial Registration:**

Dutch Trial Register NTR-6891. Retrospectively registered 28 December 2017.

## Background

Bacterial meningitis (BM) is a severe infection of the central nervous system that mainly affects infants and young children. Specifically in the Netherlands, where this study is based, estimates indicate there are ~ 205 new cases of BM in children annually [1]. The incidence of acute community-acquired BM is 0.9–2.6 per 100,000 per year in high-income countries [2, 3]. Incidence rates have dropped dramatically in the Netherlands [4] over the years, especially following the introduction of a vaccination campaign that started in 1993 that targeted *Haemophilus influenzae* type b, a major causative agent of BM in young children. Other common pathogens involved in BM are *Neisseria meningitidis* and *Streptococcus pneumoniae*. Meanwhile, *Streptococcus agalactiae* is the predominant pathogen in neonates younger than 1 month of age [3].

Children who survive BM often have severe to moderate or mild sequelae, with reported rates varying from 10 to 20% [5–8]. The most common severe sequelae are profound hearing impairment, neuromotor disabilities (e.g. spasticity), blindness, and mental retardation [5–11]. More subtle sequelae are reported in 20–30% of children who have had BM [8, 10, 12–14]. These include cognitive impairment, academic or behavioural disabilities and fine motor issues.

Previous research shows that the sequelae of BM contribute to a lower score in health-related quality of life in Dutch children [12]. Moreover, it is likely that mild disabilities due to BM in childhood have serious effects on social participation and quality of life during adulthood [12, 14–16]. Likewise, academic and behavioural limitations might result in a less rewarding career and greater prospects later in life. For example, mild disabilities may result in loss of productivity, a lower perceived health-related quality of life and might ultimately lead to an increased burden on society and the social health care system.

In general terms, investigations into the very long-term effects of BM from childhood into adulthood are limited [17]. This means general practitioners and adult survivors of childhood BM have little empirical evidence to use when considering the very long-term consequences of BM. Additionally, parents often feel that a comprehensive explanation of the possible after-effects is needed on discharge from hospital and also find uncertain prognoses difficult to handle [18]. Some of the existing studies on the long-term outcomes after BM show that deficits in verbal IQ, full-scale IQ, numeracy and functional ability are all possible outcomes [19–21]..

The current study will investigate the long-term health and quality of life outcomes following childhood BM. Specifically, the study will focus on outcomes during adolescence and young adulthood in two historical cohorts that survived childhood BM.

In addition, the study will also explore genetic variations relating to innate immunity that might influence longer-term outcomes following BM. Our research group previously found that single nucleotide polymorphisms (SNPs) in the Toll-like receptor 4 and 2 genes (*TLR4* and *TLR2*) are highly associated with susceptibility to meningococcal meningitis in children [22]. Moreover, SNPs in the genes for TLRs *2*, *4*, and *9* are highly associated with sensorineural hearing loss in survivors of childhood BM [23]. Activation and stimulation of TLRs at the site of infection results in the production of cytokines and chemokines. *TLR2* binds to protein porin B which is present on the outer membrane of *N. meningitidis* and also binds lipoteichoic acid that is localized in the cell wall of *S. pneumoniae* [24]. *TLR4* is activated by lipopolysaccharide in the outer membrane of *N. meningitidis* and pneumococcal lysin [25]. *TLR9* is an intracellular pathogen recognition receptor which recognizes CpG (non-methylated cytosine-phosphate-guanine) motifs in bacterial DNA [26]. Mutated variants may enhance susceptibility caused by hypo-responsiveness to pathogens but on the contrary, may also lead to protection if variants enhance the crucial initiation of the inflammatory response against pathogens. In order to use host genetic factors in prediction models for outcomes after BM, more genetic variants should be studied to create the best subset of variants with the strongest predictive potential. Therefore, the secondary objective of this study is to investigate genetic variants and their role in susceptibility to BM and whether they can be used to predict outcomes after BM.

## Methods/Design

### Objective

The main objective of this study is to determine whether BM in childhood is associated with impaired functioning in adolescence and young adulthood and to identify children most at risk of developing adverse outcomes. It will particularly focus on subtle sequelae and also examine host genetic factors and clinical variables.

### Key questions

During a period of 2 years the following study questions will be addressed:

1. What is the health-related quality of life and participation in society of BM survivors approximately 25 years after infection?
2. To what degree is BM in childhood associated with adverse health-related quality of life in young adulthood, when quality of life is compared to that of a general adult population?
3. To what degree is adverse quality of life (compared to a general adult population) related to the age of onset and the pathogen causing BM?
4. What is the degree of economic burden in young adulthood after childhood BM, in terms of income, health-care utilization, and absenteeism from study or work?
5. Which SNPs (alone or in a genetic trait) in immune response genes are associated with susceptibility to and severity of BM, and other related long-term outcomes?

### Setting

**Fig. 1:**
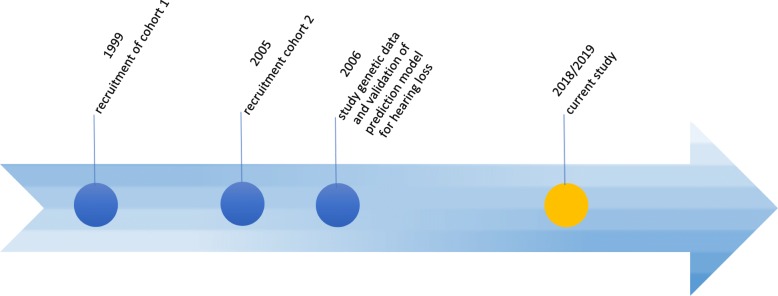
Timeline Blue circle: previous studies. Yellow circle: current study.

**Fig. 2:**
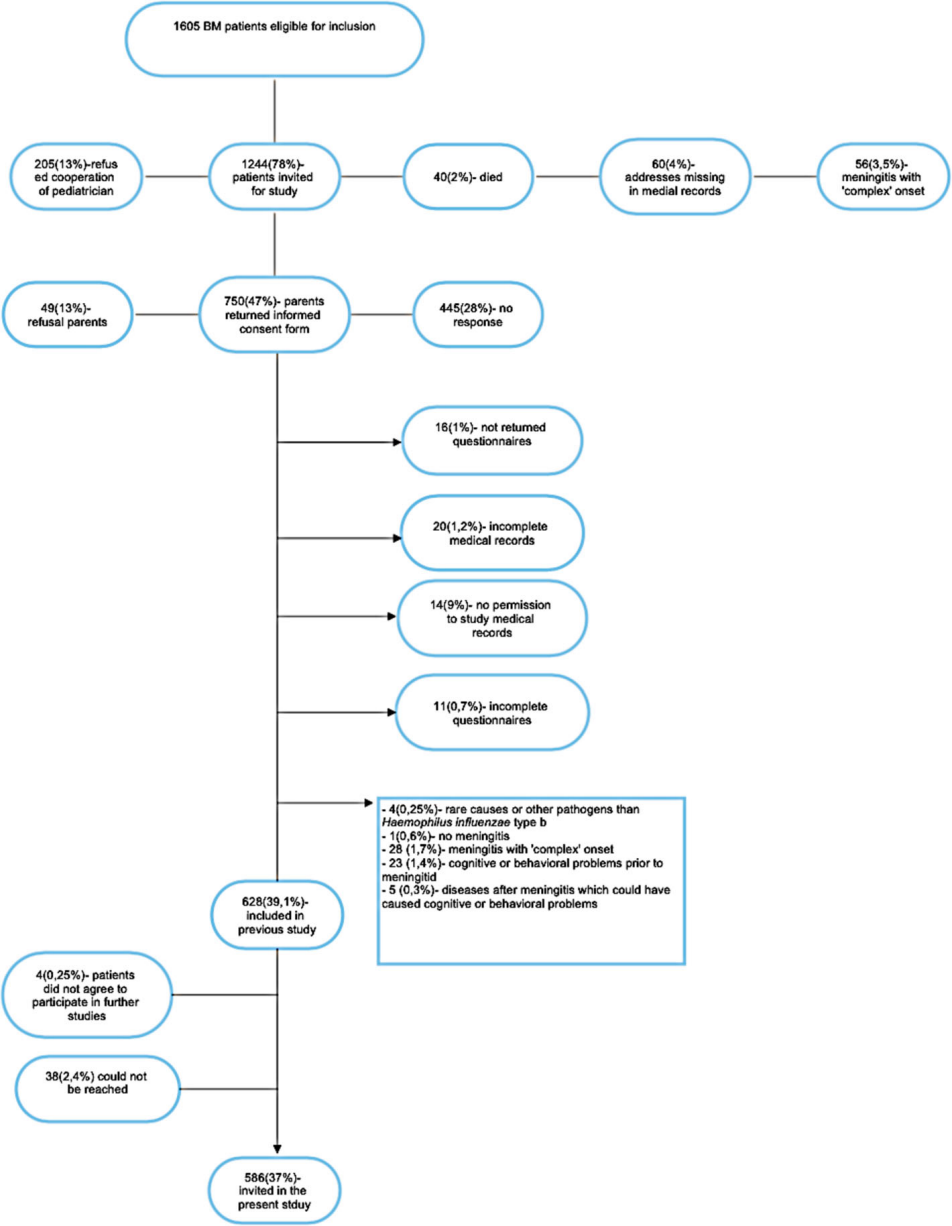
Flowchart Cohort 1

**Fig. 3:**
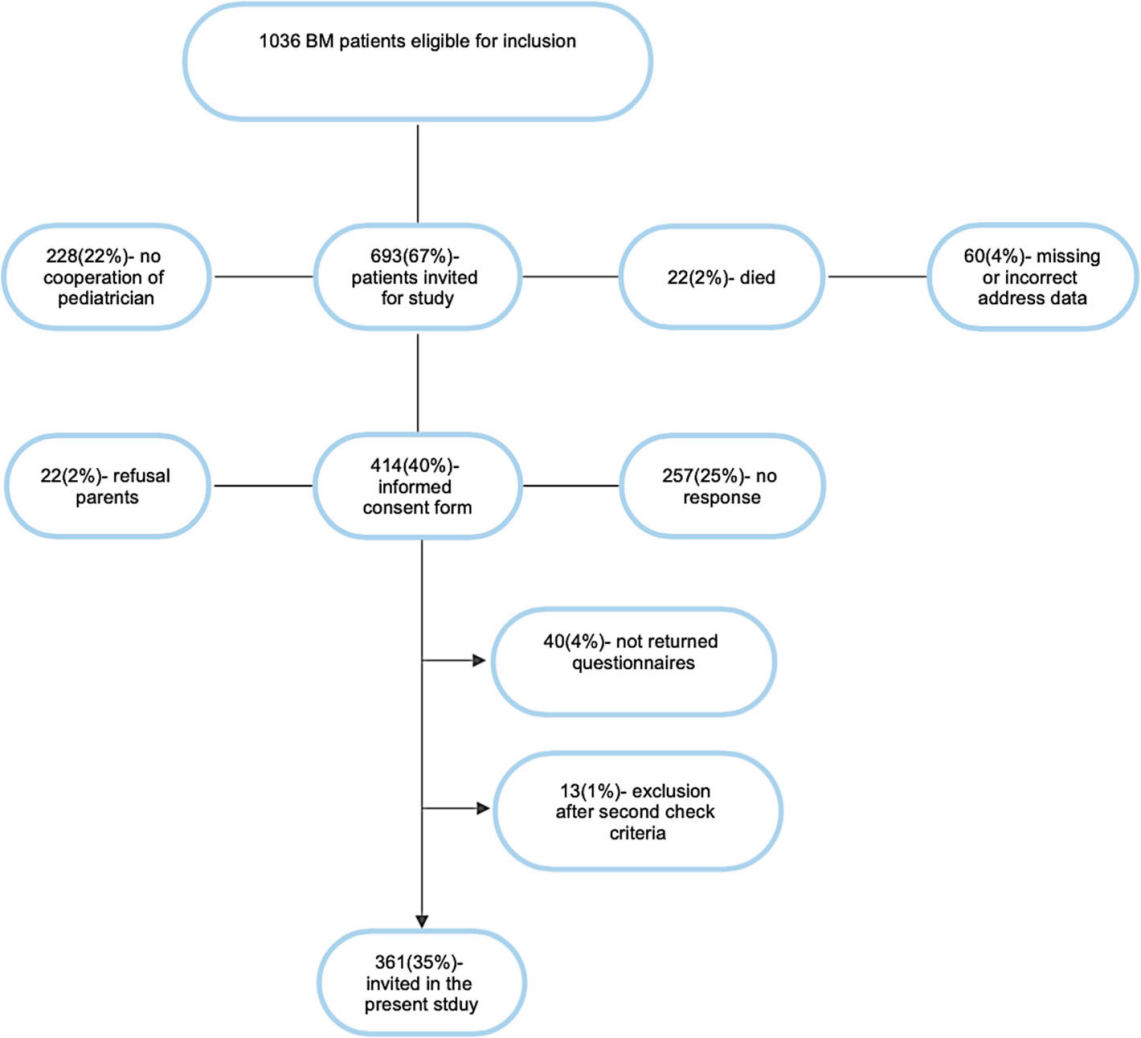
Flowchart Cohort 2

In 1999, a study was initiated in The Netherlands to determine the incidence of adverse consequences after childhood BM. This had a focus on the prediction of changes in hearing loss, academic achievement and behavioral limitations at school-age (see timeline displayed in Fig. 1). In 1999 and 2005, two cohorts of childhood BM survivors of school age were constructed as part of this study. Both cohorts are used in the present study and were retrospectively selected based on data from analyses of bacterial cerebrospinal fluid (CSF) isolates from the Netherlands Reference Laboratory for Bacterial Meningitis (NRLBM). These isolates were obtained from patients treated for BM in 110 Dutch hospitals. For the first cohort, files of the NRLBM were searched for children born between January 1986 and December 1994 who survived BM between January 1990 and December 1995 (see Fig. 2). 1605 children were eligible for inclusion and pediatricians were approached to send their parents a letter requesting participation. 628 children were included and their medical records were investigated for risk factors and for perceptive hearing loss of > 25 dB [12]. The second cohort consisted of 361 children born between January 1993 and December 1999 who suffered from non-*haemophilus* type b BM between January 1997 and December 2001 (see Fig. 3 and Table 1) [27]. This second cohort was mainly constructed for the external validation of earlier constructed prediction models for outcomes after childhood BM.

**Table 1:** Overview of meningitis cohort

Cohort 1	*N* = 586
Median age	28.0 (IQR 27.0–30.0)
Male*	335 (57)
Female*	251 (43)
Cohort 2	*N* = 361
Median age	22.0 (IQR 21.0–24.0)
Male*	200 (55)
Female*	161 (45)
Total cohort present study	*N* = 947
Median age	26.0 (IQR 22.0–28.0)
Male*	535 (56)
Female*	412 (44)

*Values represent numbers (percentages)

In 2006, both cohorts were approached to participate in a follow-up study to collect genetic data by analyzing buccal DNA. A total of 471 patients (70%) returned a buccal swab. The methodology used to construct these cohorts is extensively described in earlier published studies [12, 27, 28]. All patients from the original cohorts who consented to future follow-up studies will be invited for the present study.

### Study population

The parents of 624 patients of the first cohort agreed to participate in further studies. During the last follow-up round 38 parents could not be reached. The remaining 586 young adults will be invited to participate in the present study (see Table 1). Median age of all patients at infection was 2.1 (IQR] 0.8–3.8) years. At the time of the present study, all patients will have a median age of years 26 (IQR 22.0–28.0) (see Table 1). Participants will be censored when contact details cannot be retrieved from the municipal registers.

### Collection of information

The original study was initiated in 1999, indicating that the addresses of the patients might need to be updated in order to perform the current study. Local municipal registers will be used for this update. The patients will receive a letter in which information is stated about the relevance, procedure of the study and a link to complete the online internet-based questionnaires. The patients will be asked to sign an online informed consent form. Study subjects can revoke their informed consent to participate at any time without explanation.

At baseline, information was obtained from medical records, and in 2006, parents of children from the second cohort completed the Child Behaviour Checklist (CBCL) and the Strengths and Difficulties Questionnaires (SDQ) [12, 29]. The questionnaires in the present study will focus on the following six domains: health-related quality of life including participation in society, hearing, cognitive function, academic achievement, behavioral functioning and economic burden.

#### Health-related quality of life

To determine health-related quality of life and participation in society the following questionnaires will be used: the Global Health 10 PROMIS (Patient Reported Outcomes Measurement Information System)-29 Profile questionnaire, PROMIS Satisfaction 2.0 questionnaire and the Weiss functional Impairment Rating Scale Self-Report (WFIRS-S) [30, 31].

#### Hearing

A slightly modified used to examine subjective hearing. The adjustments included changes to questions to focus on adolescents and young adults that did not solely focus on hearing aid adjustments. Originally developed for adults [32–34], the AIADH is a self-assessment questionnaire that consists of 28 items covering all the relevant factors of disability in individual hearing functioning in daily life. AIADH has high validity and reliability, according to previous research [33].

#### Academic achievement and cognitive functioning

Academic achievement will be examined with questions related to type of school, history of school career (e.g. what kind of primary and secondary school did they attend, and did they participate in subsequent education). Executive functions will be measured by Behaviour Rating Inventory of Executive Function-Adult Version (BRIEF-A). This consists of a self-assessment and an informant list of questions which both consist of 75 items. The items cover behavioural regulation (inhibition, flexibility, regulation of emotion, self-evaluation) and metacognition (taking initiative, working memory, planning and organizing, job evaluation, orderliness) [35, 36].

#### Behavioural functioning

The Adult Self Report (ASR) is a 126-item self-report questionnaire for adults (ages 18–59) that assesses aspects of adaptive functioning and problems. The questionnaire provides scores for the following syndrome scales: anxious/depressed, withdrawn, somatic complaints, thought problems, attention problems, aggressive behavior, rule-breaking behavior, and intrusive behavior. The questionnaire provides scores for the following DSM- oriented scales: depressive problems, anxiety problems, somatic problems, avoidant personality problems, attention deficit/ hyperactivity problems (inattention and hyperactivity/impulsivity subscales), and antisocial personality problems. Additionally, the questionnaire asks about the use of the following substances: tobacco, alcohol, and drugs. The CBCL and the ASR are part of a system of comparable instruments in terms of content, administration and scoring.

#### Health-care utilization and absenteeism from study-work

The Institute for Medical Technology Assessment (iMTA) Productivity Cost Questionnaire (iPCQ) and iMTA Medical Consumption Questionnaire (iMCQ) will be used to measure the impact of BM on the ability of a person to perform work. The iMCQ is a generic instrument for measuring medical costs and includes questions relating to frequently occurring contacts with health care providers. The iPCQ measures and values productivity losses [37, 38].

### Collection of samples

Participants that complete the questionnaires will be approached to provide a swab for buccal DNA. DNA will be isolated from the buccal swabs and analysed using Kompetitive Allele specific polymerase chain reaction technology (KASP). A selection of 20–40 SNPs will be typed within and downstream the TLR signalling pathway but also within genes involved in the production of inflammation cytokines (e.g. IL-1).

### Statistical analysis

#### Data processing

Data will be assessed for the potential for bias due to non-response and the extent of missing data. Univariable comparisons of the distribution of patient characteristics and clinical data will be performed with independent one sample-t-tests for continuous data and χ^2^-test for nominal data. Fisher’s exact test will be used if the data does not meet the criteria for a χ^2^-test. In general, data will be presented as mean (standard deviation) for normally distributed variables, and as median (IQR) for continuous variables that are not normally distributed. Normality of the distribution of the data will be assessed by evaluating distribution plots and the Shapiro–Wilk test.

#### Prediction of quality of life

The results of all individual questionnaires (except iMCQ and iPCQ since these questionnaires are not validated yet) will be compared to the general population using one-sample t-tests or the one-sample Wilcoxon signed rank test, depending on normality of distribution of the data [30, 34, 36, 39]. Comparisons of quality of life health-care utilization, absenteeism, executive functioning, academic achievement and behavioural functioning will be made between cases (patients with and without hearing loss at baseline). Multiple regression analysis will be used to examine associations of functioning in daily life (WFIRS-S) with quality of life (PROMIS Global health) adjusting for the following potential confounders: birth weight, administration of dexamethasone during infection, gestational age, type of antibiotics, education level of parents, socio-economic status, current age, age of onset and severity of sequelae. All associations will be explored for differences in effect between male and female, but also for differences in effect between causal pathogens with stratified analysis and interaction tests.

#### Prediction of the course of behavioral (dis-)functioning

To determine the course of behavioral, dis-functioning, a one sample paired t-test will be performed on the second cohort as we will have data from both the ASR and the CBCL. Multiple regression analysis will be used to examine associations between behavioral functioning (ASR) and quality of life (PROMIS Global Health questionnaire) adjusting for the following potential confounders: education level of parents, school results and executive functioning. All associations will be explored for differences in effect between male and female, but also for differences in effect between causal pathogens with stratified analysis and interaction tests.

#### Analysis of genetic data

Combined carriage of two or more SNPs will also be investigated to determine whether certain traits are associated with outcomes after BM. The lasso method will be used to determine the potential predictive value of SNPs and to assess the association with susceptibility. This is a statistical method to reliably select variables when there are more variables than outcome categories (also called the events per variable problem) [40]. Regression analysis will be used to assess significant differences, while hearing will be used as an outcome variable. All results will be considered statistically significant if a two-sided *p*-value of < 0.05 is reached. The Hardy-Weinberg equilibrium will be applied to assess Mendelian inheritance.

## Discussion

During the Dutch 20|30 Postmeningitis study, the very long-term effects after childhood BM will be investigated in two historical cohorts with an individual follow-up time of up to ~ 25 years. The questionnaires will provide data on health-related quality of life including participation in society, hearing, cognitive functioning, academic achievement, behavioural functioning, economic self-sufficiency, and utilization of medical care. More insight into long-term outcomes after childhood BM is needed to better align the follow-up of these children and will also enable clinicians to inform parents and address important long-term consequences of childhood BM. Children at high risk of developing sequelae should be identified at an early stage after experiencing BM in order to provide timely interventions or prevent complications. Single nucleotide polymorphisms seem a valuable addition to clinical prediction models [41, 42]. By further exploring genetic variation in patients with BM the best set of variants with the strongest predictive potential can be assembled.

### Strengths

The 20|30 Dutch Postmeningitis study will be the first to investigate quality of life after childhood BM in historical cohorts with a follow-up of approximately 25 years. The follow-up duration in combination with the several domains assessed with questionnaires should make this study valuable from multiple perspectives. In particular, economic self-sufficiency and medical treatment use at such a long outlook have not been investigated. As such, this study will provide new information on very long-term outcomes after BM and potentially guide the clinical follow-up of children who experience BM. There is no consensus currently on which children should be followed over time and for how long. Clearly, clinicians will keep observing and treating children with severe sequelae. However, in the case of children with more subtle sequelae the picture is less clear when considering longer term outcomes. Extended information on the course of mild sequelae after BM would be beneficial from a clinical perspective. Specifically, knowledge of risk factors that contribute to long-term care after BM would also be valuable.

### Limitations

First of all, the genetic domains that we will investigate should be carefully considered as associations with childhood BM because of the cross-sectional study design and the fact that this study lacks a control group. To determine possible long-term adverse effects of childhood BM in young adulthood it would be better to study all domains at several moments in time. Furthermore, there is a risk of selection bias as patients with less or more sequelae might be more motivated to participate in this study. There is also a risk that participation with the online questionnaires might limit the participation of patients with more profound sequelae. In addition, both historical cohorts used in this study had an overall difference in age and were not treated at the same time.

### In summary

BM is still a life-threatening infectious disease and this study will provide more insight into the very long-term outcomes which could have a significant impact on subsequent health state of childhood BM survivors during young adult life. Prediction of the outcomes using host genetic risk factors and clinical variables may contribute to better understanding, timely intervention and thereby appropriate follow-up of children after BM.

## Data Availability

The datasets that have been used and that will be used during the current study are available from the corresponding author upon reasonable request.

## Abbreviations

ADHD: Attention Deficit Hyperactivity Disorder
AIADH: Amsterdam Inventory for Auditory Disability and Handicap
ASR: Adult Self Report
BM: Bacterial Meningitis
BRIEF-A: Behaviour Rating Inventory of Executive Function-Adult Version
CBCL: Child Behaviour Checklist
DNA: Deoxyribonucleic acid
iMCQ: iMTA Medical Consumption Questionnaire
iMTA: Institute for Medical Technology Assessment
iPCQ: iMTA Productivity Cost Questionnaire
NRLBM: Netherlands Reference Laboratory for Bacterial Meningitis
PROMIS: Patient Reported Outcomes Measurement Information System
SD: Standard Deviation
SNP: Single Nucleotide Polymorphism TLR Toll-like receptor
WFIRS-S: Weiss functional Impairment Rating Scale Self-Report
